# Differently Expressed Circular RNAs in Lacrimal Sacs From Patients With Chronic Dacryocystitis

**DOI:** 10.3389/fgene.2022.834111

**Published:** 2022-02-28

**Authors:** Yue Li, Xueru Liu, Wenyue Zhang, Xuefei Song, Leilei Zhang, Caiwen Xiao

**Affiliations:** ^1^ Department of Ophthalmology, School of Medicine, Shanghai Ninth People’s Hospital, Shanghai Jiao Tong University, Shanghai, China; ^2^ Shanghai Key Laboratory of Orbital Diseases and Ocular Oncology, Shanghai, China

**Keywords:** chronic dacryocystitis, CirRNA, competing endogenous RNA, RNA sequnce, pathogenesis

## Abstract

This study was designed to identify differently expressed circular RNAs (circRNAs) and investigate their potential roles in lacrimal sacs from patients with chronic dacryocystitis. The lacrimal sac samples of three chronic dacryocystitis patients and three control subjects were collected for RNA sequencing after ribosomal RNA was depleted. Differently expressed circRNAs and messenger RNAs (mRNAs) were used for co-expression analysis. CircRNA-microRNA (miRNA)-mRNA interaction network were also established by miRanda software. Meanwhile, pathway and functional enrichment analysis were conducted for the down- and up-regulated mRNAs in the circRNA-mRNA co-expression network. The expression levels of circRNAs and mRNAs in chronic dacryocystitis and control samples were validated by quantitative real-time reverse transcription polymerase chain reaction (qRT-PCR). In all the 3,909 circRNAs predicted through RNA sequencing, 25 circRNAs (20 up-regulated and 5 down-regulated) expressed differently in chronic dacryocystitis samples. Besides, there identified 1,486 differentially expressed mRNAs. Of these differently expressed circRNAs and mRNAs, eight were validated by qRT-PCR, including MYH2, DSP, CD27, CCL5, FN1, has_circ_0004792, has_circ_0001062, and has_circ_0115476. Gene Ontology (GO) analysis indicated that the majority of altered mRNAs in this co-expression network were involved in immune system processes and meanwhile Kyoto Encyclopedia of Genes and Genomes (KEGG) analysis revealed that these altered expressed mRNAs were also amplified in bacterial invasion of epithelial cells, both of which were thought to be involved in the pathogenesis of chronic dacryocystitis. In the circRNA-miRNA-mRNA interaction network, six circRNAs were found to be related to Th1 and Th2 cell differentiation, which was closely associated with the development of chronic dacryocystitis. This study identified statistically significant differences between circRNAs and mRNAs of lacrimal sac samples of chronic dacryocystitis patients and control individuals and provides novel insight into the regulatory mechanism of circRNAs, miRNAs, and mRNAs in the pathogenesis of chronic dacryocystitis.

## Introduction

Chronic dacryocystitis is the most common disorder in the lacrimal duct system and causes approximately 3% of total ophthalmologic clinic visits in polyclinic (Amin et al., 2013; Avdagic and Phelps 2020). Characterized by the symptoms of persistent epiphora and mucopurulent discharge, chronic dacryocystitis seriously affects patients’ quality of life (Taylor and Ashurst 2021). What’s worse, although itself a relatively benign condition, chronic dacryocystitis, is known to alter the conjunctival flora of affected eyes. Then the altered flora can form the nidus for vision-threatening infections following intraocular surgery or any other breach in ocular surface (Mitra et al., 2019). As for its treatment, dacryocystorhinostomy is considered the standard procedure for treating chronic dacryocystitis but it’s invasive and has a risk of recurrence (Chen and Liu 2019; Ullrich et al., 2021). In addition, the incidence of chronic dacryocystitis is still growing, emphasizing the urgent need to clarify its pathogenesis and to work out effective treatment measures (Badhu et al., 2005; Costea et al., 2017; Chen et al., 2018).

However, despite the above threaten and high incidence of chronic dacryocystitis, its pathogenesis has still not been fully understood. It has been acknowledged to be closely related to obstruction of the nasolacrimal duct, accumulation of desquamated cells, pooling of tears, as well as secretions and mucus upstream of the obstruction, all of which create a fertile environment to allow bacterial colonization and promotes inflammation of the lacrimal sac (Luo et al., 2021; Perez et al., 2021). Recently more and more researchers are trying to further elucidate the pathogenesis of chronic dacryocystitis and have proved some other factors such as the absorption function of the nasolacrimal duct and immune factors also take part in the progression of chronic dacryocystitis. There was lacrimal drainage–associated lymphoid tissue (LDALT), which contains typical lymphoid follicles or scattered lymphocytes and constitutes part of the mucosa-associated lymphoid tissue (MALT) in the lacrimal duct (Knop and Knop 2001). Experiments have shown that in the lacrimal sacs of chronic dacryocystitis, LDALT has changed a lot characterized by an increase in the number of a predominance of B lymphocytes and IgA-secreting plasma cells and this suggests a good immune response in chronic dacryocystitis (Ali et al., 2013). Distribution patterns of T cell subsets were also different in the lacrimal sac mucosa. Between the two subsets of T cells in LDALT of chronic dacryocystitis patients, CD4^+^ T cells were more abundant than CD8^+^ T cells. Besides, both real-time PCR and immunohistochemical staining demonstrated the expression levels of IFN-γ were significantly higher than IL-4, revealing a predominant Th1 response in chronic dacryocystitis (Yang et al., 2018a). It suggested an imbalance of Th1/Th2 status might play a role in the pathogenesis of chronic dacryocystitis. But as a whole, knowledge on molecular regulation mechanism of the occurrence and development of the disease is very limited and there was no research on the pathogenesis of chronic dacryocystitis by case-control gene and protein expression analysis.

CircRNA was a specific RNA more stable than long non-coding RNAs and has a closed circular structure (Zhou et al., 2020b). Recent studies have revealed that circRNA molecules may act as competing endogenous RNAs (ceRNAs). They are rich in miRNA binding sites, making them an miRNA sponge in cells and thus relieving the inhibition of miRNA on its target genes and then expression levels of these target genes increase (Kulcheski et al., 2016; Chen et al., 2020; Wu et al., 2020). Many circRNAs exert important biological functions through regulating protein function, through acting as microRNA or protein inhibitors (“sponges”), or by being translated themselves (Chen 2020). Furthermore, circRNAs have been implicated in lots of diseases such as neurological disorders, diabetes mellitus, cardiovascular diseases, and cancer (Kristensen et al., 2019). CircRNAs have also been reported in many ocular diseases such as diabetic retinopathy, glaucoma, dry eye disease, thyroid-associated ophthalmopathy and age-related macular degeneration (Liu et al., 2020; Wu et al., 2020; Su et al., 2021). However, there is no reports on high-throughput sequencing of circRNA expression profiling in chronic dacryocystitis. Hence, the expression profile and the clinical significance of circRNAs in chronic dacryocystitis and the potential mechanisms that cause the occurrence and development of the disease need to be further investigated.

In this study, we conducted high-throughput RNA sequencing for lacrimal sac samples from chronic dacryocystitis patients and control subjects to identify differentially expressed circRNAs and mRNAs. Afterwards, circRNA-miRNA-mRNA interaction and circRNA-mRNA co-expression networks were constructed to reveal the potential roles of differently expressed circRNAs in the pathogenesis of chronic dacryocystitis. What’s more, differentially expressed mRNAs and circRNAs were validated using qRT-PCR.

## Material and Methods

### Ethical Approval

This study followed the tenets of the Declaration of Helsinki and was approved by the Ethics Committee of Shanghai Ninth People’s Hospital. Written informed consents were obtained from all the patients.

### Patients and Tissue Samples

Lacrimal sac samples of 9 patients diagnosed as chronic dacryocystitis were collected from the wastes during endoscopic dacryocystorhinostomy (En-DCR) surgery. The diagnosis of chronic dacryocystitis relied on the symptoms of epiphora and discharge, assessment of the lacrimal drainage system, including inspection, palpation of the lacrimal sac, diagnostic lacrimal duct irrigation, and computed tomography dacryocystography (CT-DCG) when necessary. Symptoms lasted longer than 6 months were regarded chronic. Patients with glaucoma, cataracts, and other ocular disease or a serious systemic disease were excluded. Patients suffered from systemic inflammatory or auto- immune diseases or received immunosuppressive agents and/or systemic or local steroids within 6 months were also excluded. Meanwhile, lacrimal sac samples of 9 control individuals were obtained from the wastes during dacryocystectomy for orbital trauma patients. All the control subjects had no history of systemic or local inflammatory and autoimmune diseases or any other ocular disorders. All participants had not received any eye drops or lacrimal duct irrigation 1 month before surgery.

### RNA Extraction and Sequencing Analysis

Total RNA was extracted from lacrimal sac samples of chronic dacryocystitis patients and control individuals using Trizol (Invitrogen, Carlsbad, CA, United States) following the manufacturer’s instructions. The quality and quantity of total RNA were measured on the Nanodrop 2000 (Thermo Scientific, Wilmington, DE). Electrophoresis of a denaturing agarose gel was used to assess RNA integrity.

Then ribosomal RNA (rRNA) was depleted and strand-specific RNA-seq libraries were built with KAPA Stranded RNA-Seq Library Prep Kit (Illumina) according to the manufacturer’s instructions. Then Agilent 2,100 Bioanalyzer (Agilent Technologies, Santa Clara, CA, United States) was used to perform library quality control. High-throughput RNA sequencing was performed on the Illumina NovaSeq 6,000 platform (Illumina, San Diego, CA), and using 150bp paired-end reads were produced.

### Differential Expression Analysis and circRNA-mRNA Co-Expression Network Construction

With *p* value <0.05 and |log2 FC (fold change) | ≥ 1 as the cut-off criteria, DESeq package was applied to screen differentially expressed mRNAs and circRNAs. In the meantime, pheatmap package in R was used to conduct hierarchical clustering analysis for all samples on the basis of expressions levels of identified mRNAs and circRNAs (Version 1.0.8). Co-expression analysis was performed on the basis of the expression levels of differentially expressed circRNAs and mRNAs in chronic dacryocystitis and normal control samples. Using Cytoscape software, correlated pairs of circRNAs and mRNAs with *p* value <0.05 and a Pearson’s correlation coefficient >0.98 were selected to construct the circRNA-mRNA co-expression network (Version 3.2.1).

### CircRNA-miRNA-mRNA Regulatory Network Construction

TargetScan and miRanda were used to predict the circRNA-miRNA interactions and the target gene of miRNAs. Expression correlations between circRNAs, mRNAs and miRNAs with a threshold of Pearson’s correlation coefficient >0.85 or <−0.85 and *p* value <0.05 were filtered out. Then, the circRNA-ceRNA regulatory networks were constructed using the Cytoscape software (Version 3.2.1).

### Functional Analysis for mRNAs in Co-Expression Networks

With a cut-off criterion of *p* value <0.05, biological process (BP), molecular function (MF), cellular component (CC) in GO analysis, and potential pathways in KEGG analysis were performed to study the function of mRNAs with differential expression levels in the constructed circRNA-miRNA-mRNA and circRNA-mRNA interaction networks.

### Validation of Expression Levels of circRNAs and mRNAs by qRT-PCR

Total RNA was extracted from lacrimal sac samples from 6 chronic dacryocystitis patients and 6 control individuals using Trizol (Invitrogen, Carlsbad, CA, United States) following the manufacturer’s instructions. Using the PrimeScript RT reagent Kit (Takara Bio Company, Shiga, Japan), the RNA was reverse-transcribed into cDNA and the cDNA was amplified on an ABIPRISM 7500 Sequence Detection System (Applied Biosystems, Foster City, United States) using the SYBR Green Kit (Takara Bio Company). The housekeeping gene GAPDH was used as an internal reference and a control for PCR product quantification and normalization. To evaluate the relative expression levels of target genes, the 2^−ΔΔCt^ method was applied. The primers used in our study for circRNAs and mRNAs were synthesized by Tsingke (Shanghai, China). The primer sequences were listed in Table 1.

**TABLE 1:** Primer sequences for quantitative real-time polymerase chain reaction (qRT-PCR) analysis.

Gene symbol	Forward primer	Reverse primer	Product length (bp)
CD27	TGC​AGA​GCC​TTG​TCG​TTA​CAG	GCT​CCG​GTT​TTC​GGT​AAT​CCT	83
CCL5	TGC​TGC​TTT​GCC​TAC​ATT​GC	CTT​GTT​CAG​CCG​GGA​GTC​AT	168
MYH2	CTG​AGG​GAG​GAG​CGA​CTC​T	CTC​GGG​CTT​ATA​CAC​AGG​CA	224
DSP	GGA​GAA​CCT​TGG​TTG​GCA​GA	TTC​ATA​TTC​TCT​CTT​CCG​GGT​G	118
FN1	CGG​TGG​CTG​TCA​GTC​AAA​G	AAA​CCT​CGG​CTT​CCT​CCA​TAA	130
GAPDH	GGA​GCG​AGA​TCC​CTC​CAA​AAT	GGC​TGT​TGT​CAT​ACT​TCT​CAT​GG	197
has_circ_0115476	CAC​CCG​AAC​ACC​ATT​CAC​AG	TTT​CTA​GCT​CCT​CGT​CCG​TC	181
has_circ_0004792	CGG​GAG​GAG​AAT​GAC​AAG​GA	TCA​CGT​TCT​TCT​GAT​GGC​CT	162
has_circ_0001062	TCC​AAA​AGG​AGA​AAA​CGT​GAG​A	TCT​GCT​TTT​CAT​TCT​CTT​TTG​CT	119

## Results

### Representative Clinical Characteristics of Chronic Dacryocystitis Patients

**FIGURE 1:**
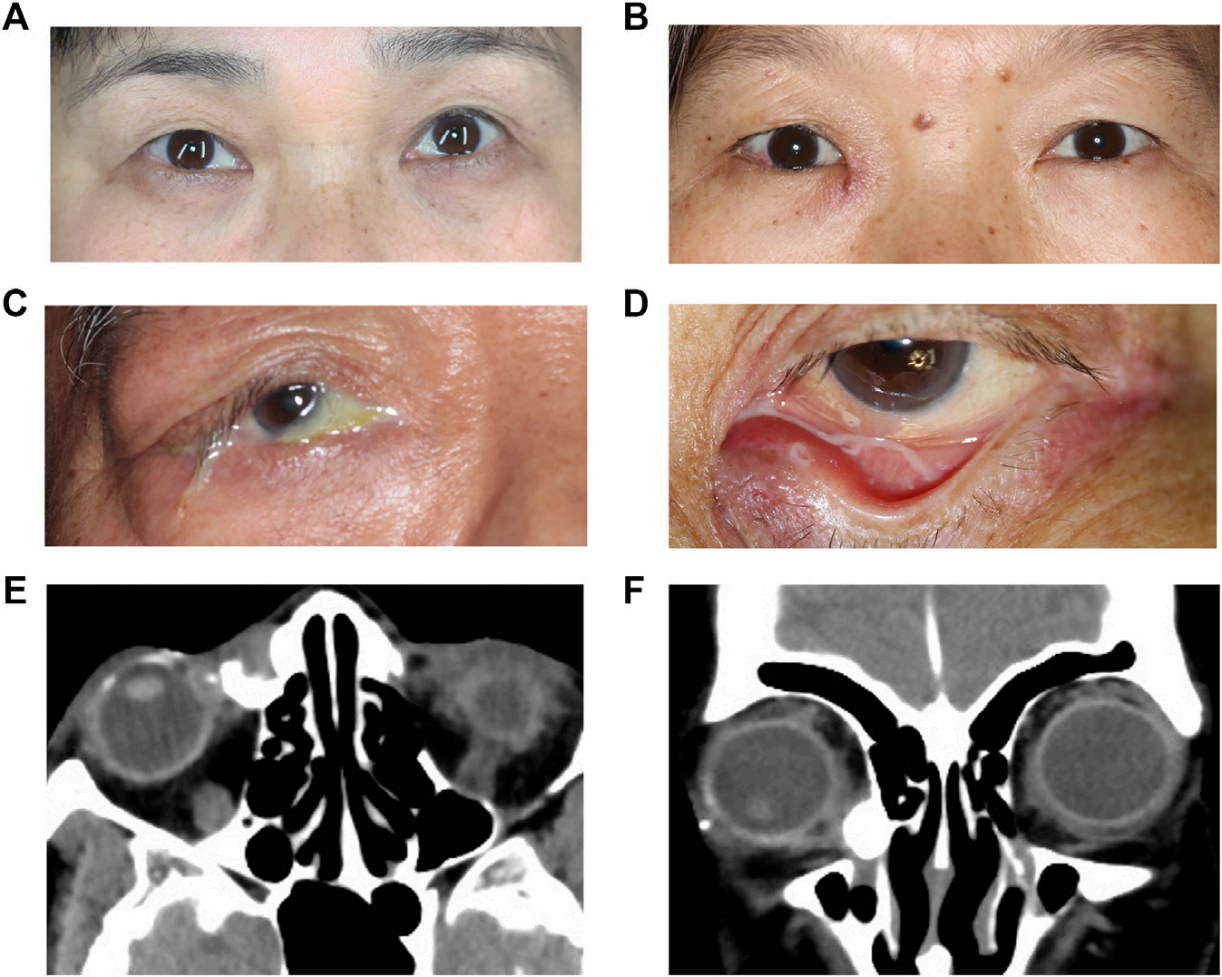
Clinical manifestation of chronic dacryocystitis patients. **(A–D)** Representative clinical characteristics of patients with chronic dacryocystitis. **(E,F)** Representative computed tomography-dacryocystography (CT-DCG) images of patients with chronic dacryocystitis.

In this study, 9 lacrimal sac samples of chronic dacryocystitis patients and 9 samples of control individuals were collected, of which 3 pairs were used for sequencing and the other 6 pairs were used for qRT-PCR to validate the sequencing in each group. The demographic data of chronic dacryocystitis and control groups is shown in Table 2. There were 6 women and 3 men in chronic dacryocystitis group while in control group (orbital trauma patients) there were 5 women and 4 men, but there was no statistically significant difference in gender between these two groups (*p* = 0.234, χ2 test). The average age was 52.3 ± 6.6 years in chronic dacryocystitis group and 50.3 ± 4.3 years in control group, but there was also no significant difference between these two groups (*p* = 0.458, independent *t*-test). Representative pictures of the patient’s appearance are shown in Figures 1A–D. Featured by persistent epiphora and mucopurulent discharge, chronic dacryocystitis patients tend to have eczema around inner canthus (Figures 1A, B). Moreover, in some severe conditions, there is empyemata in the conjunctival sac or the whole ocular surface (Figures 1C, D), which has a risk of intraocular infection and hinders the performance of ophthalmic surgeries. Representative CT-DCG images of patients with chronic dacryocystitis were shown in Figures 1E, F.

**TABLE 2:** Demographic data of chronic dacryocystitis and control groups.

Case	Age (years)	Gender	Disease	Side	Lacrimal duct irrigation within 1 month	Eye drops within 1 month
1	44	F	Chronic dacryocystitis	L	No	No
2	61	F	Chronic dacryocystitis	R	No	No
3	50	M	Chronic dacryocystitis	R	No	No
4	51	F	Chronic dacryocystitis	L	No	No
5	44	M	Chronic dacryocystitis	L	No	No
6	53	F	Chronic dacryocystitis	L	No	No
7	50	M	Chronic dacryocystitis	L	No	No
8	55	F	Chronic dacryocystitis	R	No	No
9	63	F	Chronic dacryocystitis	R	No	No
10	50	F	Orbital trauma	R	No	No
11	54	F	Orbital trauma	R	No	No
12	53	M	Orbital trauma	L	No	No
13	53	M	Orbital trauma	R	No	No
14	45	F	Orbital trauma	L	No	No
15	55	F	Orbital trauma	R	No	No
16	53	M	Orbital trauma	L	No	No
17	47	M	Orbital trauma	L	No	No
18	43	F	Orbital trauma	R	No	No

F, female; M, male; L, left eye; R, right eye; mean age, 52.3 ± 6.6 (chronic dacryocystitis), 50.3 ± 4.3 (orbital trauma).

### Identification and Characterization of circRNAs

**FIGURE 2:**
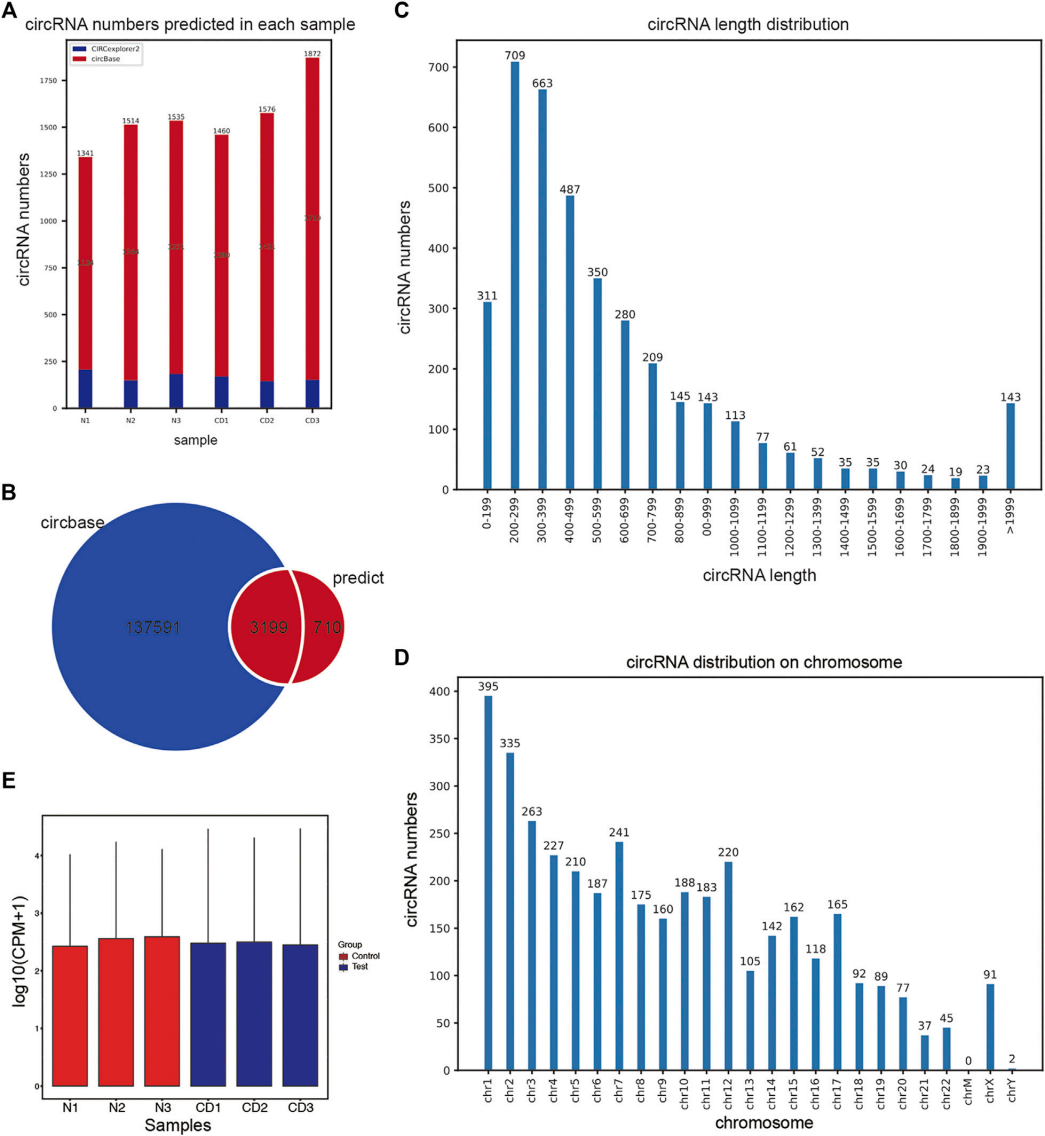
Prediction of circRNAs in the lacrimal sac samples through high-throughput RNA sequencing. **(A)** The number of circRNAs predicted in lacrimal sac samples from chronic dacryocystitis patients (CD1, CD2, and CD3) and control individuals (N1, N2, and N3). **(B)** Venn diagram for predicted circRNAs. **(C)** CircRNA length distribution. **(D)** CircRNA chromosome distribution. **(E)** Box plots of counts of exon model per million mapped reads (CPM) values of circRNAs in each lacrimal sac sample.

For the lacrimal sac samples from 3 chronic dacryocystitis patients and 3 control individuals, 3,909 circRNAs were predicted from RNA sequencing data. We compared our data with the data published in the circBase and found 710 (710/3,909; 18.2%) of the predicted circRNAs were novel while the other 3,199 (3,199/3,909; 81.8%) predicted circRNAs have been reported in the circBase (Figures 2A, B). The length of predicted circRNAs ranged from less than 200 nucleotides (nt) to more than 1999 nt, and most circRNAs (3,766/3,909; 96.3%) were less than 2000 nt in length (Figure 2C). These circRNAs were distributed among the entire genome but mainly in the chromosomes 1, 2, and 7 (Figure 2D). Moreover, calculated CPM (counts of exon model per million mapped reads) values were used to quantify the expression levels of circRNAs and the box-plots of CPM value of circRNAs showed there was no significant difference in the expression level of the circRNAs in each sample (Figure 2E).

### Identification of Differentially Expressed RNAs

**FIGURE 3:**
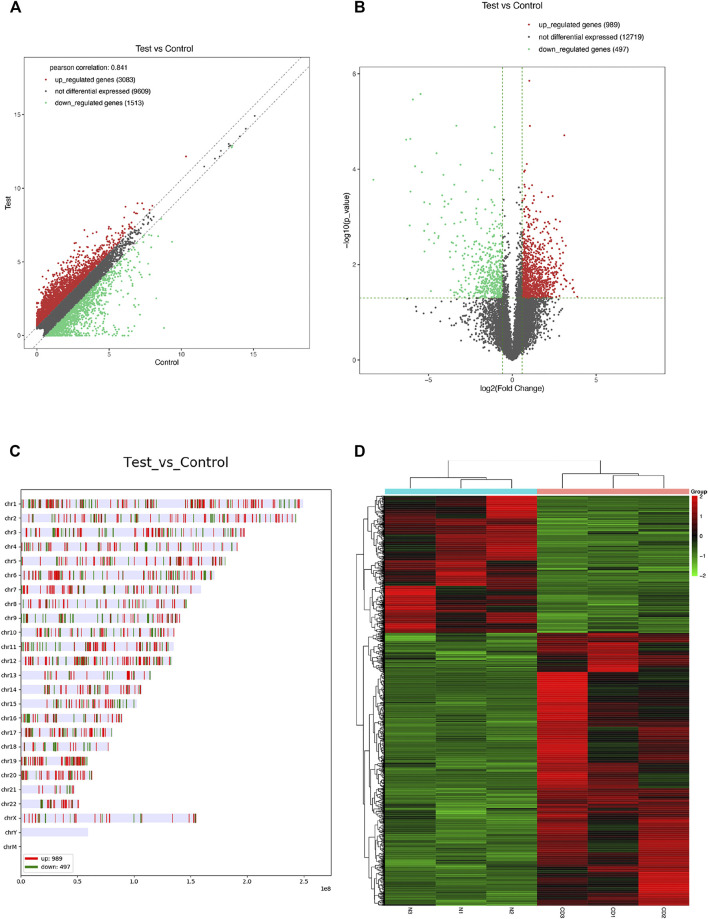
mRNA expression profiles in chronic dacryocystitis and control groups. **(A,B)** Differentially expressed mRNAs between in lacrimal sac samples from chronic dacryocystitis patients (CD1, CD2, and CD3) and control individuals (N1, N2, and N3). **(C)** Chromosome distribution of differently expressed mRNAs. **(D)** Hierarchical clustering analysis of differentially expressed mRNAs in chronic dacryocystitis and normal lacrimal sac samples.

**FIGURE 4:**
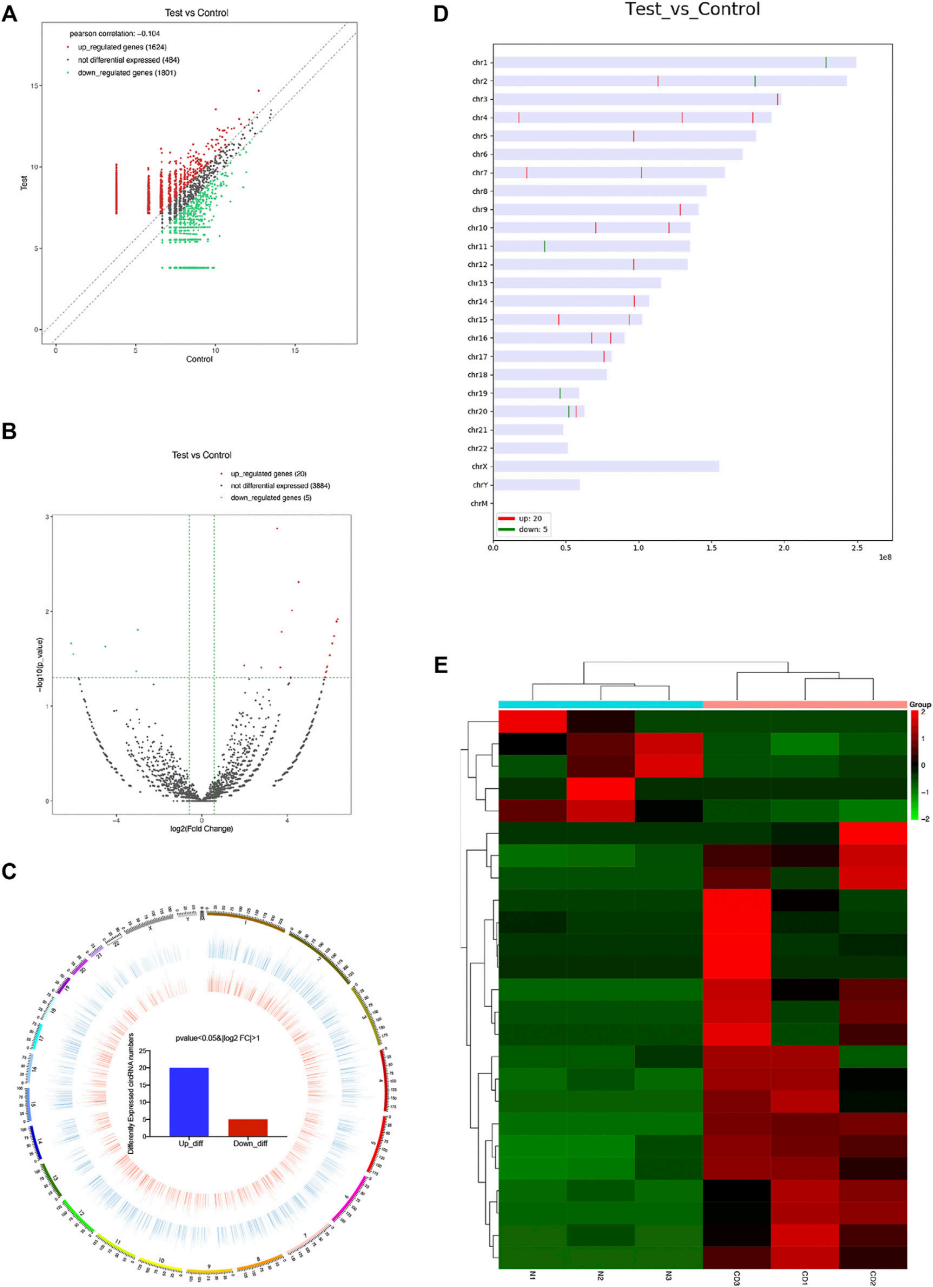
CircRNA expression profiles in chronic dacryocystitis and control groups. **(A,B)** Differentially expressed circRNAs between in lacrimal sac samples from chronic dacryocystitis patients (CD1, CD2, and CD3) and control individuals (N1, N2, and N3). **(C,D)** Chromosome distribution of up- and down-regulated circRNAs. **(E)** Hierarchical clustering analysis of differentially expressed circRNAs in chronic dacryocystitis and normal lacrimal sac samples.

With a thresholds of |log2 FC| ≥ 1 and *p* value <0.05, we analyzed the differentially expressed mRNAs and circRNAs. RNA-seq analysis revealed a total of 1,486 differentially expressed mRNAs (989 up-regulated and 497 down-regulated) between chronic dacryocystitis samples and control samples (Figures 3A, B; Supplementary Table S1). Distribution of differently expressed mRNAs was also shown in Figure 3C. In addition, hierarchical clustering analysis revealed that based on differentially expressed mRNAs, chronic dacryocystitis samples could be distinguished from the control samples (Figure 3D). Meanwhile, there were 25 differentially expressed circRNAs (20 up-regulated and 5 down-regulated) identified through RNA sequencing (Figures 4A, B; Supplementary Table S2). Of the 20 up-regulated circRNAs, 17 were reported previously and 3 were novel. Meanwhile, of the 5 down-regulated circRNAs, 2 were recorded in the circBase before and 3 were newly discovered. Distribution of differently expressed circRNAs on chromosomes was also described in Figures 4C, D. Hierarchical clustering analysis demonstrated that chronic dacryocystitis samples was distinguished from the control subjects depending on differentially expressed circRNAs (Figure 4E). The results indicated that there was significant difference in mRNA and circRNA expression profiles between lacrimal sacs of chronic dacryocystitis patients and control individuals.

### Construction of circRNA-mRNA Interaction Network and Functional Analyses of Dysregulated mRNAs in This Network

**FIGURE 5:**
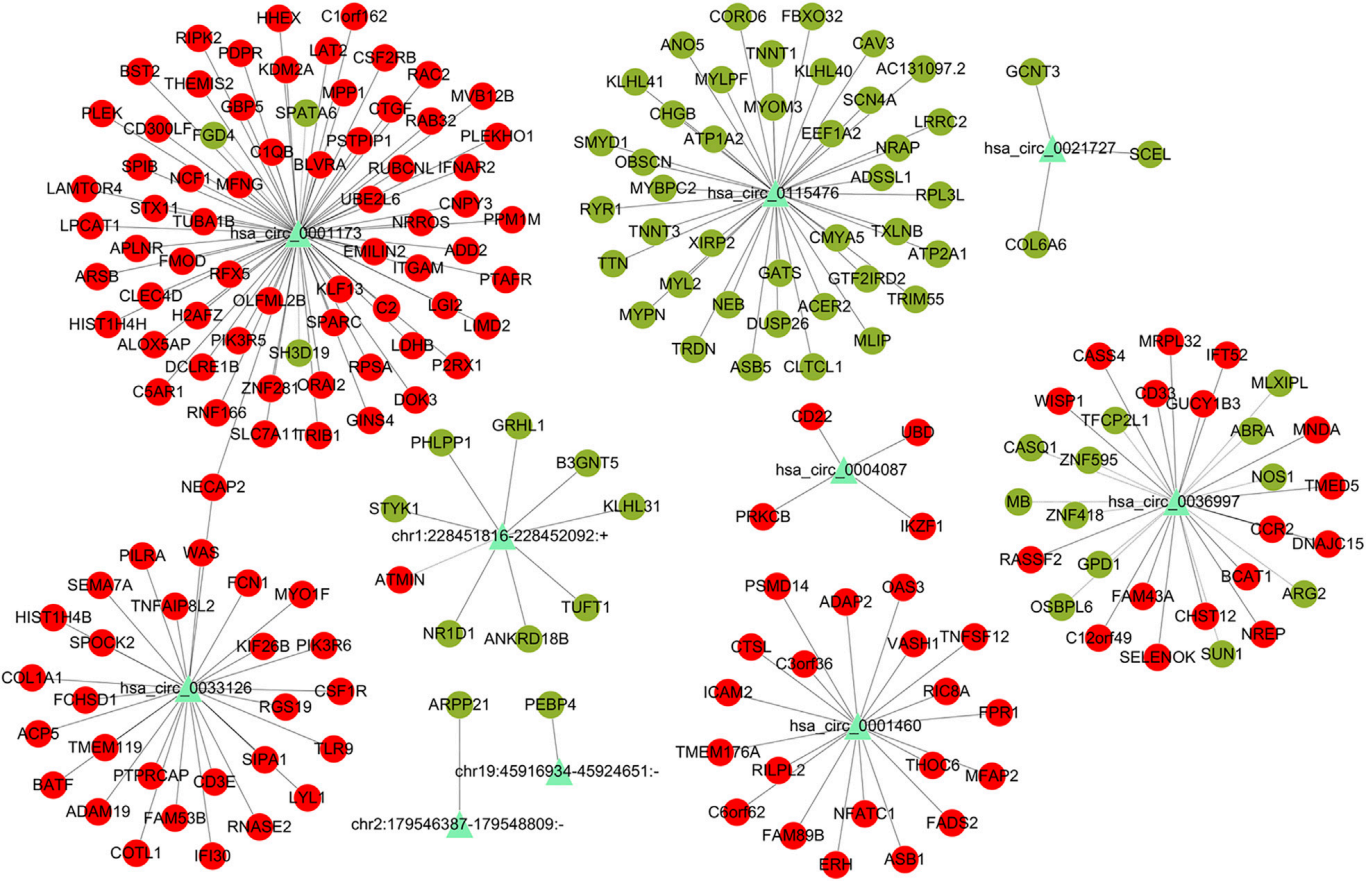
CircRNA-mRNA co-expression network of differentially expressed mRNAs and dysregulated circRNAs. Triangles represent dysregulated circRNAs. Round dots in red represent up-regulated mRNAs, round dots in green represent down-regulated mRNAs. Black lines represent correlations between circRNAs and mRNAs.

**FIGURE 6:**
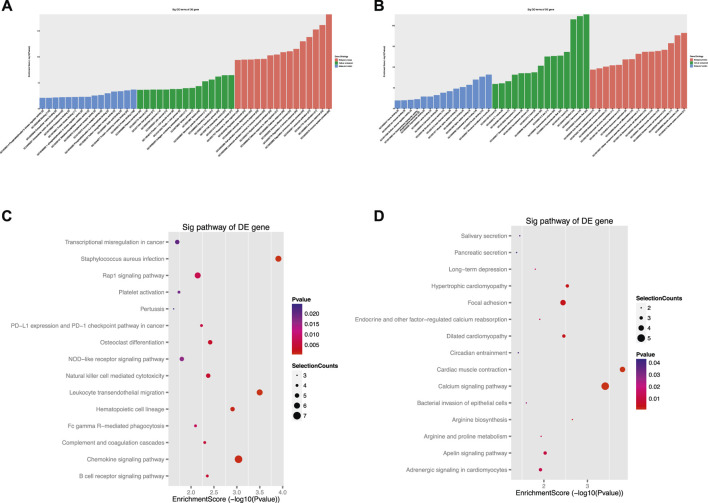
Gene Ontology (GO) and Kyoto Encyclopedia of Genes and Genomes (KEGG) analysis for mRNAs in the circRNA-mRNA co-expression network. **(A, B)** The top 45 GO terms of biological process (BP), cellular component (CC) and molecular function (MF) categories for up-regulated mRNAs **(A)** and down-regulated mRNAs **(B)**. **(C,D)** The top 15 KEGG pathways of upregulated mRNAs **(C)** and downregulated mRNAs **(D)**.

To further investigate the mechanisms underlying the functions of the identified differently circRNAs, we constructed circRNA associated regulatory networks. First, we constructed circRNA-mRNA interaction network. Top 5 of up-regulated and top 3 of down-regulated circRNAs were selected and co-expression analysis was performed depending on the expression levels of selected circRNAs and differentially expressed mRNAs to calculate *p* value and the Pearson’s correlation coefficient. The co-expression network was conducted by Cytoscape 3.2.1 using correlated pairs between circRNAs and mRNAs with *p* value <0.05 and a Pearson’s correlation coefficient >0.98 (Figure 5). To further explore potential functions of mRNAs involved in the circRNA-mRNA interaction network, GO and KEGG analysis were performed. The GO functional enrichment analysis indicated up-regulated mRNAs in the circRNA-mRNA co-expression network were mainly associated with the BP of immune system process (such as ITGAM and C5AR1), CC of cytoplasmic vesicle (such as MVB12B and TLR9), and MF of protein binding (such as STX11 and NFATC1) (Figure 6A; Table 3). The down-regulated mRNAs were chiefly associated with the BP of muscle system process (such as TNNT1 and MYOM3), CC of contractile fiber (such as MYLPF), and MF of structural constituent of muscle (such as NEB and OBSCN) (Figure 6B; Table 3). Results of KEGG pathway enrichment analysis revealed that most of the up-regulated mRNAs were involved in the Fc gamma R-mediated phagocytosis (such as NCF1, PRKCB, RAC2, and WAS), chemokine signaling pathway (such as CCR2, NCF1, PIK3R5, and PIK3R6) and *Staphylococcus aureus* infection (such as C5AR1 and FPR1) (Figure 6C; Table 4). The down-regulated mRNAs mainly participated in the calcium signaling pathway (such as ATP2A1 and CASQ1) and bacterial invasion of epithelial cells (such as CAV3 and CLTCL1) (Figure 6D; Table 4).

**TABLE 3:** The top 30 Gene Ontology (GO) terms for up- and down-regulated mRNAs in the circRNA-mRNA co-expression network in biological process (BP), cellular component (CC), and molecular function (MF) categories.

mRNAs	ID	Term	Category	Count	*p* Value	Enrichment score	Gene
Up-regulated	GO:0002376	Immune system process	BP	65	6.71E-19	18.17	ITGAM; C5AR1; FCN1; MNDA; PSMD14; CLEC4D; ICAM2; RNASE2; SELENOK; CSF1R; PLEK; CTSL; PRKCB; LAT2; RIPK2; CD3E; MFNG; BATF; CCR2; FPR1; NCF1; WAS; TLR9; BST2; CD33; NRROS; PTAFR; SEMA7A; TNFSF12; C1QB; C2; RAB32; IFI30; ADD2; IKZF1; HHEX; LYL1; MMP9; CD22; CD300LF; SLC7A11; UBD; NFATC1; RAC2; PIK3R6; COTL1; ARSB; P2RX1; LPCAT1; DOK3; CNPY3; RNF166; TNFAIP8L2; PSTPIP1; GBP5; TRIB1; KLF13; RASSF2; COL1A1; PILRA; THEMIS2; IFNAR2; OAS3; MPP1; TMEM176A
	GO:0006955	Immune response	BP	52	7.70E-17	16.11	FCN1; MNDA; PSMD14; CLEC4D; ICAM2; RNASE2; CTSL; PRKCB; LAT2; RIPK2; CD3E; MFNG; FPR1; C5AR1; WAS; CCR2; BST2; CD33; C1QB; C2; UBD; NFATC1; PIK3R6; RAC2; COTL1; ITGAM; ARSB; MMP9; P2RX1; PTAFR; LPCAT1; DOK3; BATF; CNPY3; RNF166; CSF1R; TLR9; NCF1; TNFAIP8L2; PSTPIP1; GBP5; COL1A1; CD300LF; PILRA; CD22; THEMIS2; IFI30; OAS3; IFNAR2; SEMA7A; NRROS; TNFSF12
	GO:0001775	Cell activation	BP	41	5.45E-16	15.26	ITGAM; C5AR1; CLEC4D; MFNG; PLEK; IKZF1; COL1A1; PIK3R6; PIK3R5; P2RX1; PRKCB; HHEX; LYL1; CD3E; CD22; MNDA; TLR9; CD300LF; RIPK2; SELENOK; WAS; BST2; LAT2; RAC2; UBD; BATF; CCR2; PSMD14; FCN1; COTL1; FPR1; ARSB; MMP9; PTAFR; RNASE2; LPCAT1; DOK3; CD33; CTSL; TNFAIP8L2; SLC7A11
	GO:0045321	Leukocyte activation	BP	37	1.54E-14	13.81	ITGAM; C5AR1; CLEC4D; MFNG; IKZF1; HHEX; LYL1; CD3E; CD22; MNDA; TLR9; CD300LF; RIPK2; SELENOK; WAS; PRKCB; BST2; LAT2; RAC2; UBD; BATF; CCR2; PSMD14; FCN1; COTL1; FPR1; ARSB; MMP9; P2RX1; PTAFR; RNASE2; LPCAT1; DOK3; CD33; CTSL; PIK3R6; TNFAIP8L2
	GO:0002682	Regulation of immune system process	BP	40	1.04E-13	12.98	FCN1; MNDA; PSMD14; CLEC4D; ICAM2; SELENOK; FPR1; C5AR1; WAS; TLR9; CD3E; CCR2; BST2; CD33; C1QB; C2; CSF1R; CD22; CD300LF; SLC7A11; RIPK2; NFATC1; LAT2; RAC2; PIK3R6; ITGAM; PTAFR; GBP5; PRKCB; TRIB1; KLF13; RASSF2; COL1A1; PILRA; THEMIS2; TNFAIP8L2; IFNAR2; SEMA7A; MPP1; TMEM176A
	GO:0050776	Regulation of immune response	BP	30	2.97E-12	11.53	FCN1; MNDA; PSMD14; CLEC4D; ICAM2; FPR1; C5AR1; WAS; CCR2; BST2; CD33; C1QB; C2; NFATC1; LAT2; PIK3R6; RAC2; ITGAM; PTAFR; GBP5; RIPK2; CD3E; THEMIS2; PRKCB; CD22; IFNAR2; SEMA7A; COL1A1; CD300LF; PILRA
	GO:0002252	Immune effector process	BP	33	8.50E-12	11.07	FCN1; SELENOK; CLEC4D; MFNG; WAS; TLR9; CCR2; BST2; C1QB; C2; C5AR1; PIK3R6; LAT2; RAC2; PSMD14; COTL1; FPR1; ITGAM; ARSB; MMP9; MNDA; P2RX1; PTAFR; RNASE2; LPCAT1; DOK3; CD33; BATF; RIPK2; CD22; IFNAR2; OAS3; SEMA7A
	GO:0006952	Defense response	BP	39	1.29E-11	10.89	ITGAM; C5AR1; FCN1; MNDA; PSMD14; CLEC4D; ICAM2; RNASE2; SELENOK; P2RX1; GBP5; CSF1R; FPR1; NRROS; TLR9; PTAFR; CCR2; SEMA7A; RIPK2; PSTPIP1; THEMIS2; NCF1; UBD; BST2; PIK3R6; BATF; CNPY3; RNF166; C1QB; C2; TNFAIP8L2; ACP5; COTL1; IFNAR2; OAS3; IFI30; WAS; MMP9; PTPRCAP
	GO:0098542	Defense response to other organism	BP	31	3.67E-11	10.43	FCN1; MNDA; PSMD14; CLEC4D; ICAM2; RNASE2; SELENOK; UBD; BST2; PIK3R6; TLR9; BATF; CNPY3; RNF166; CSF1R; ITGAM; NCF1; C1QB; C2; TNFAIP8L2; RIPK2; PSTPIP1; GBP5; ACP5; C5AR1; COTL1; IFNAR2; OAS3; IFI30; PTAFR; WAS
	GO:0002274	Myeloid leukocyte activation	BP	23	5.25E-11	10.28	ITGAM; C5AR1; CLEC4D; CD300LF; UBD; BATF; LAT2; RAC2; CCR2; PSMD14; FCN1; COTL1; FPR1; ARSB; MMP9; MNDA; P2RX1; PTAFR; RNASE2; BST2; LPCAT1; DOK3; CD33
	GO:0031410	Cytoplasmic vesicle	CC	37	3.33E-07	6.48	MVB12B; TLR9; RAB32; CD22; CTSL; BST2; STX11; NECAP2; SIPA1; TMED5; RGS19; COL1A1; FPR1; P2RX1; PTAFR; C5AR1; DOK3; RAC2; SPARC; NCF1; PSMD14; FCN1; COTL1; LPCAT1; ARSB; MNDA; RNASE2; CLEC4D; ITGAM; CD33; WAS; FCHSD1; LAMTOR4; MMP9; GBP5; ADD2; RUBCNL
	GO:0097708	Intracellular vesicle	CC	37	3.47E-07	6.46	MVB12B; TLR9; RAB32; CD22; CTSL; BST2; STX11; NECAP2; SIPA1; TMED5; RGS19; COL1A1; FPR1; P2RX1; PTAFR; C5AR1; DOK3; RAC2; SPARC; GBP5; ADD2; RUBCNL; NCF1; PSMD14; FCN1; COTL1; LPCAT1; ARSB; MNDA; RNASE2; CLEC4D; ITGAM; CD33; WAS; FCHSD1; LAMTOR4; MMP9
	GO:0030141	Secretory granule	CC	20	6.41E-07	6.19	FPR1; P2RX1; PTAFR; C5AR1; DOK3; SPARC; PSMD14; FCN1; COTL1; BST2; LPCAT1; ARSB; MNDA; RNASE2; CLEC4D; ITGAM; CD33; CTSL; MMP9; COL1A1
	GO:0099503	Secretory vesicle	CC	21	2.40E-06	5.62	STX11; COL1A1; FPR1; P2RX1; PTAFR; C5AR1; DOK3; SPARC; PSMD14; FCN1; COTL1; BST2; LPCAT1; ARSB; MNDA; RNASE2; CLEC4D; ITGAM; CD33; CTSL; MMP9
	GO:0030667	Secretory granule membrane	CC	11	5.11E-06	5.29	SPARC; FPR1; BST2; LPCAT1; CLEC4D; ITGAM; P2RX1; CD33; PTAFR; DOK3; C5AR1
	GO:0031982	Vesicle	CC	46	4.40E-05	4.36	MVB12B; TLR9; RAB32; CD22; CTSL; BST2; STX11; WAS; NECAP2; SIPA1; TMED5; RGS19; COL1A1; FPR1; P2RX1; PTAFR; C5AR1; DOK3; RAC2; SPARC; GBP5; ADD2; RUBCNL; NCF1; PSMD14; FCN1; COTL1; LPCAT1; ARSB; MNDA; RNASE2; CLEC4D; ITGAM; CD33; FCHSD1; PILRA; RPSA; LDHB; MMP9; PRKCB; BLVRA; C2; LAT2; LAMTOR4; MYO1F; RIPK2
	GO:0070820	Tertiary granule	CC	7	9.86E-05	4.01	CLEC4D; ITGAM; PTAFR; CD33; FPR1; DOK3; MMP9
	GO:0062023	Collagen-containing extracellular matrix	CC	11	1.09E-04	3.96	COL1A1; SPARC; CTSL; FCN1; FMOD; MFAP2; MMP9; C1QB; EMILIN2; SEMA7A; ADAM19
	GO:0101002	Ficolin-1-rich granule	CC	6	1.59E-04	3.80	PSMD14; FCN1; COTL1; ARSB; MMP9; MNDA
	GO:1904813	Ficolin-1-rich granule lumen	CC	6	1.59E-04	3.80	PSMD14; FCN1; COTL1; ARSB; MMP9; MNDA
	GO:0005515	Protein binding	MF	109	2.04E-04	3.69	STX11; NFATC1; FAM89B; FCN1; FPR1; ITGAM; C5AR1; RGS19; RIC8A; CTSL; COL1A1; ADD2; VASH1; COTL1; MYO1F; WAS; RPSA; PLEK; PRKCB; LAMTOR4; CNPY3; TNFSF12; RIPK2; CD22; CD300LF; TLR9; ICAM2; SEMA7A; KIF26B; IFT52; SIPA1; PSTPIP1; TRIB1; HHEX; NCF1; ADAM19; CD3E; IFNAR2; RAC2; CSF1R; CD33; IKZF1; CCR2; RAB32; TUBA1B; UBE2L6; CLEC4D; LAT2; PILRA; GBP5; RILPL2; LDHB; MMP9; P2RX1; SELENOK; BCAT1; BST2; DCLRE1B; LYL1; ALOX5AP; NRROS; PTAFR; CASS4; PSMD14; IFI30; UBD; BATF; RNF166; FAM43A; CSF2RB; PIK3R6; APLNR; ERH; KDM2A; ATMIN; ZNF281; SLC7A11; DNAJC15; MFAP2; MNDA; MPP1; OAS3; TMED5; PLEKHO1; KLF13; ASB1; TMEM176A; ADAP2; PTPRCAP; RFX5; RNASE2; BLVRA; MRPL32; SPARC; C1QB; C2; TNFAIP8L2; DOK3; C3ORF36; RUBCNL; ORAI2; LIMD2; EMILIN2; GINS4; MVB12B; NREP; THEMIS2; RASSF2; SPOCK2
	GO:0044877	Protein-containing complex binding	MF	20	2.87E-04	3.54	CD22; ICAM2; ITGAM; SEMA7A; CTSL; MMP9; SPARC; ADD2; PIK3R5; RAB32; CD3E; RPSA; COTL1; MYO1F; PSTPIP1; PSMD14; UBD; ATMIN; P2RX1; DCLRE1B
	GO:0033691	Sialic acid binding	MF	3	4.22E-04	3.37	FCN1; CD22; CD33
	GO:0038187	Pattern recognition receptor activity	MF	3	4.83E-04	3.32	PTAFR; FCN1; TLR9
	GO:0043325	Phosphatidylinositol-3,4-bisphosphate binding	MF	3	1.07E-03	2.97	PLEK; ADAP2; NCF1
	GO:0140375	Immune receptor activity	MF	5	2.24E-03	2.65	FPR1; C5AR1; IFNAR2; CSF2RB; CCR2
	GO:0004875	Complement receptor activity	MF	2	2.86E-03	2.54	C5AR1; FPR1
	GO:0008191	Metalloendopeptidase inhibitor activity	MF	2	5.11E-03	2.29	BST2; SPOCK2
	GO:0046935	1-phosphatidylinositol-3-kinase regulator activity	MF	2	5.11E-03	2.29	PIK3R6; PIK3R5
	GO:0019901	Protein kinase binding	MF	11	5.55E-03	2.26	PLEK; PRKCB; TRIB1; NFATC1; PTAFR; CASS4; ADD2; IFNAR2; RAC2; WAS; CD3E
Down-regulated	GO:0003012	Muscle system process	BP	21	5.78E-19	18.24	TNNT1; TNNT3; TTN; TRDN; MYOM3; MYLPF; NOS1; RYR1; SCN4A; CAV3; ATP1A2; KLHL41; ARG2; MYL2; MLIP; CASQ1; FBXO32; MYBPC2; NEB; MB; ATP2A1
	GO:0006936	Muscle contraction	BP	19	2.12E-18	17.67	TNNT1; TNNT3; ATP1A2; KLHL41; ARG2; NOS1; TTN; MYL2; CASQ1; CAV3; MYBPC2; NEB; MB; ATP2A1; SCN4A; TRDN; MYOM3; MYLPF; RYR1
	GO:0030239	Myofibril assembly	BP	11	1.74E-16	15.76	TTN; KLHL41; TNNT1; TNNT3; OBSCN; CASQ1; MYPN; MYL2; CAV3; NEB; NRAP
	GO:0055002	Striated muscle cell development	BP	13	6.78E-15	14.17	KLHL41; TTN; TNNT1; TNNT3; OBSCN; CASQ1; MYPN; KLHL40; RYR1; MYL2; NEB; NRAP; CAV3
	GO:0006941	Striated muscle contraction	BP	13	1.34E-14	13.87	TNNT1; TNNT3; MYL2; ATP1A2; CASQ1; CAV3; MB; ATP2A1; NOS1; TTN; SCN4A; KLHL41; ARG2
	GO:0055001	Muscle cell development	BP	13	1.92E-14	13.72	KLHL41; TTN; CAV3; TNNT1; TNNT3; OBSCN; CASQ1; MYPN; KLHL40; RYR1; MYL2; NEB; NRAP
	GO:0051146	Striated muscle cell differentiation	BP	15	2.12E-14	13.67	NOS1; CAV3; SMYD1; KLHL41; TTN; TNNT1; TNNT3; OBSCN; CASQ1; MYPN; KLHL40; RYR1; MYL2; NEB; NRAP
	GO:0010927	Cellular component assembly involved in morphogenesis	BP	11	6.37E-14	13.20	KLHL41; TTN; TNNT1; TNNT3; OBSCN; CASQ1; MYPN; MYL2; CAV3; NEB; NRAP
	GO:0042692	Muscle cell differentiation	BP	15	1.22E-12	11.91	NOS1; CAV3; SMYD1; KLHL41; TTN; MYL2; TNNT1; TNNT3; OBSCN; CASQ1; MYPN; KLHL40; RYR1; NEB; NRAP
	GO:0045214	Sarcomere organization	BP	8	1.46E-12	11.84	CAV3; KLHL41; TNNT1; TNNT3; TTN; OBSCN; CASQ1; MYPN
	GO:0043292	Contractile fiber	CC	20	1.92E-23	22.72	MYLPF; TNNT1; TNNT3; MYL2; OBSCN; MYOM3; ABRA; FBXO32; XIRP2; NEB; NOS1; NRAP; RYR1; TTN; CASQ1; MYPN; CAV3; KLHL41; CMYA5; KLHL40
	GO:0030017	Sarcomere	CC	19	5.17E-23	22.29	TNNT1; TNNT3; FBXO32; XIRP2; NEB; NOS1; NRAP; RYR1; TTN; OBSCN; CASQ1; MYPN; CAV3; KLHL41; MYOM3; CMYA5; KLHL40; MYL2; ABRA
	GO:0030016	Myofibril	CC	19	3.09E-22	21.51	TNNT1; TNNT3; MYOM3; ABRA; MYL2; FBXO32; XIRP2; NEB; NOS1; NRAP; RYR1; TTN; OBSCN; CASQ1; MYPN; CAV3; KLHL41; CMYA5; KLHL40
	GO:0031674	I band	CC	12	2.19E-14	13.66	FBXO32; XIRP2; NEB; NOS1; NRAP; RYR1; TTN; OBSCN; CASQ1; MYPN; CAV3; KLHL40
	GO:0099512	Supramolecular fiber	CC	22	1.77E-13	12.75	MYLPF; TNNT1; TNNT3; TRIM55; MYL2; OBSCN; MYOM3; ABRA; FBXO32; XIRP2; NEB; NOS1; NRAP; RYR1; TTN; CASQ1; MYPN; CAV3; KLHL41; CMYA5; KLHL40; MYBPC2
	GO:0099081	Supramolecular polymer	CC	22	2.08E-13	12.68	MYLPF; TNNT1; TNNT3; TRIM55; MYL2; OBSCN; MYOM3; ABRA; FBXO32; XIRP2; NEB; NOS1; NRAP; RYR1; TTN; CASQ1; MYPN; CAV3; KLHL41; CMYA5; KLHL40; MYBPC2
	GO:0030018	Z disc	CC	11	2.81E-13	12.55	FBXO32; XIRP2; NEB; NOS1; NRAP; RYR1; TTN; OBSCN; CASQ1; MYPN; CAV3
	GO:0099080	Supramolecular complex	CC	22	4.74E-11	10.32	MYLPF; TNNT1; TNNT3; TRIM55; MYL2; OBSCN; MYOM3; ABRA; FBXO32; XIRP2; NEB; NOS1; NRAP; RYR1; TTN; CASQ1; MYPN; CAV3; KLHL41; CMYA5; KLHL40; MYBPC2
	GO:0031672	A band	CC	6	1.88E-09	8.73	KLHL41; MYOM3; CMYA5; OBSCN; KLHL40; MYL2
	GO:0016529	Sarcoplasmic reticulum	CC	7	2.89E-09	8.54	TRDN; RYR1; CASQ1; KLHL41; ATP2A1; NOS1; CMYA5
	GO:0008307	Structural constituent of muscle	MF	6	6.42E-09	8.19	MYLPF; MYBPC2; MYL2; NEB; TTN; OBSCN
	GO:0003779	Actin binding	MF	12	2.10E-08	7.68	MYL2; MYOM3; XIRP2; NEB; NRAP; TTN; CORO6; FGD4; ABRA; MYBPC2; TNNT3; MYPN
	GO:0008092	Cytoskeletal protein binding	MF	16	1.04E-07	6.98	FGD4; ABRA; MYBPC2; NRAP; TNNT3; MYPN; MYL2; TNNT1; OBSCN; TTN; SPATA6; CAV3; MYOM3; XIRP2; NEB; CORO6
	GO:0051393	Alpha-actinin binding	MF	4	1.95E-06	5.71	NRAP; TTN; MYPN; XIRP2
	GO:0042805	Actinin binding	MF	4	6.41E-06	5.19	NRAP; TTN; MYPN; XIRP2
	GO:0051371	Muscle alpha-actinin binding	MF	3	1.62E-05	4.79	NRAP; TTN; MYPN
	GO:0051015	Actin filament binding	MF	6	6.77E-05	4.17	MYOM3; XIRP2; NEB; NRAP; TTN; CORO6
	GO:0005198	Structural molecule activity	MF	10	1.54E-04	3.81	RPL3L; MYLPF; MYBPC2; MYL2; NEB; TTN; OBSCN; COL6A6; TUFT1; CLTCL1
	GO:0005516	Calmodulin binding	MF	5	5.72E-04	3.24	ARPP21; NOS1; RYR1; TTN; OBSCN
	GO:0005521	Lamin binding	MF	2	1.31E-03	2.88	SUN1; MLIP

**TABLE 4:** The top 15 enriched Kyoto Encyclopedia of Genes and Genomes (KEGG) pathways for the up- and down-regulated mRNAs in the circRNA-mRNA co-expression network.

mRNAs	ID	Term	Count	Selection counts	Fisher-P value	Enrichment score	Gene
Up-regulated	hsa05150	*Staphylococcus aureus* infection - *Homo sapiens* (human)	96	6	1.24E-04	3.91	C1QB; C2; C5AR1; FPR1; ITGAM; PTAFR
	hsa04670	Leukocyte transendothelial migration - *Homo sapiens* (human)	114	6	3.17E-04	3.50	ITGAM; MMP9; NCF1; PRKCB; RAC2; SIPA1
	hsa04062	Chemokine signaling pathway - *Homo sapiens* (human)	192	7	9.19E-04	3.04	CCR2; NCF1; PIK3R5; PIK3R6; PRKCB; RAC2; WAS
	hsa04640	Hematopoietic cell lineage - *Homo sapiens* (human)	99	5	1.24E-03	2.91	CD22; CD33; CD3E; CSF1R; ITGAM
	hsa04380	Osteoclast differentiation - *Homo sapiens* (human)	128	5	3.83E-03	2.42	ACP5; CSF1R; IFNAR2; NCF1; NFATC1
	hsa04650	Natural killer cell mediated cytotoxicity - *Homo sapiens* (human)	131	5	4.23E-03	2.37	ICAM2; IFNAR2; NFATC1; PRKCB; RAC2
	hsa04662	B cell receptor signaling pathway - *Homo sapiens* (human)	82	4	4.43E-03	2.35	CD22; NFATC1; PRKCB; RAC2
	hsa04610	Complement and coagulation cascades - *Homo sapiens* (human)	85	4	5.03E-03	2.30	C1QB; C2; C5AR1; ITGAM
	hsa05235	PD-L1 expression and PD-1 checkpoint pathway in cancer - *Homo sapiens* (human)	89	4	5.92E-03	2.23	BATF; CD3E; NFATC1; TLR9
	hsa04015	Rap1 signaling pathway - *Homo sapiens* (human)	210	6	7.17E-03	2.14	CSF1R; FPR1; ITGAM; PRKCB; RAC2; SIPA1
	hsa04666	Fc gamma R-mediated phagocytosis - *Homo sapiens* (human)	97	4	8.00E-03	2.10	NCF1; PRKCB; RAC2; WAS
	hsa04621	NOD-like receptor signaling pathway - *Homo sapiens* (human)	181	5	1.59E-02	1.80	GBP5; IFNAR2; OAS3; PSTPIP1; RIPK2
	hsa04611	Platelet activation - *Homo sapiens* (human)	124	4	1.84E-02	1.74	COL1A1; P2RX1; PIK3R5; PIK3R6
	hsa05202	Transcriptional misregulation in cancer - *Homo sapiens* (human)	192	5	2.01E-02	1.70	CSF1R; HHEX; ITGAM; LYL1; MMP9
	hsa05133	Pertussis - *Homo sapiens* (human)	76	3	2.40E-02	1.62	C1QB; C2; ITGAM
Down-regulated	hsa04260	Cardiac muscle contraction - *Homo sapiens* (human)	87	4	1.57E-04	3.80	ATP1A2; ATP2A1; MYL2; TRDN
	hsa04020	Calcium signaling pathway - *Homo sapiens* (human)	201	5	3.92E-04	3.41	ATP2A1; CASQ1; NOS1; RYR1; TRDN
	hsa00220	Arginine biosynthesis - *Homo sapiens* (human)	22	2	2.22E-03	2.65	ARG2; NOS1
	hsa05410	Hypertrophic cardiomyopathy - *Homo sapiens* (human)	90	3	2.90E-03	2.54	ATP2A1; MYL2; TTN
	hsa05414	Dilated cardiomyopathy - *Homo sapiens* (human)	96	3	3.48E-03	2.46	ATP2A1; MYL2; TTN
	hsa04510	Focal adhesion - *Homo sapiens* (human)	201	4	3.63E-03	2.44	CAV3; COL6A6; MYL2; MYLPF
	hsa04371	Apelin signaling pathway - *Homo sapiens* (human)	137	3	9.35E-03	2.03	MYL2; NOS1; RYR1
	hsa00330	Arginine and proline metabolism - *Homo sapiens* (human)	51	2	1.16E-02	1.94	ARG2; NOS1
	hsa04261	Adrenergic signaling in cardiomyocytes - *Homo sapiens* (human)	150	3	1.20E-02	1.92	ATP1A2; ATP2A1; MYL2
	hsa04961	Endocrine and other factor-regulated calcium reabsorption - *Homo sapiens* (human)	53	2	1.24E-02	1.91	ATP1A2; CLTCL1
	hsa04730	Long-term depression - *Homo sapiens* (human)	60	2	1.58E-02	1.80	NOS1; RYR1
	hsa05100	Bacterial invasion of epithelial cells - *Homo sapiens* (human)	77	2	2.52E-02	1.60	CAV3; CLTCL1
	hsa04970	Salivary secretion - *Homo sapiens* (human)	93	2	3.57E-02	1.45	ATP1A2; NOS1
	hsa04713	Circadian entrainment - *Homo sapiens* (human)	97	2	3.85E-02	1.41	NOS1; RYR1
	hsa04972	Pancreatic secretion - *Homo sapiens* (human)	102	2	4.22E-02	1.37	ATP1A2; ATP2A1

Lists of abbreviations: circRNA, circular RNAs; mRNA, messenger RNA; miRNA, microRNA; GO, gene ontology; BP, biological process; CC, cellular component; MF, molecular function; KEGG, kyoto encyclopedia of genes and genomes; qRT-PCR, quantitative real-time PCR; ceRNA, competing endogenous RNA; LDALT, lacrimal drainage–associated lymphoid tissue; MALT, mucosa-associated lymphoid tissue; En-DCR, endoscopic dacryocystorhinostomy; CT-DCG, computed tomography dacryocystography; CPM, counts of exon model per million mapped reads.

### Construction of circRNA-miRNA-mRNA Interaction Network and Functional Analyses of Dysregulated mRNAs in This Network

**FIGURE 7:**
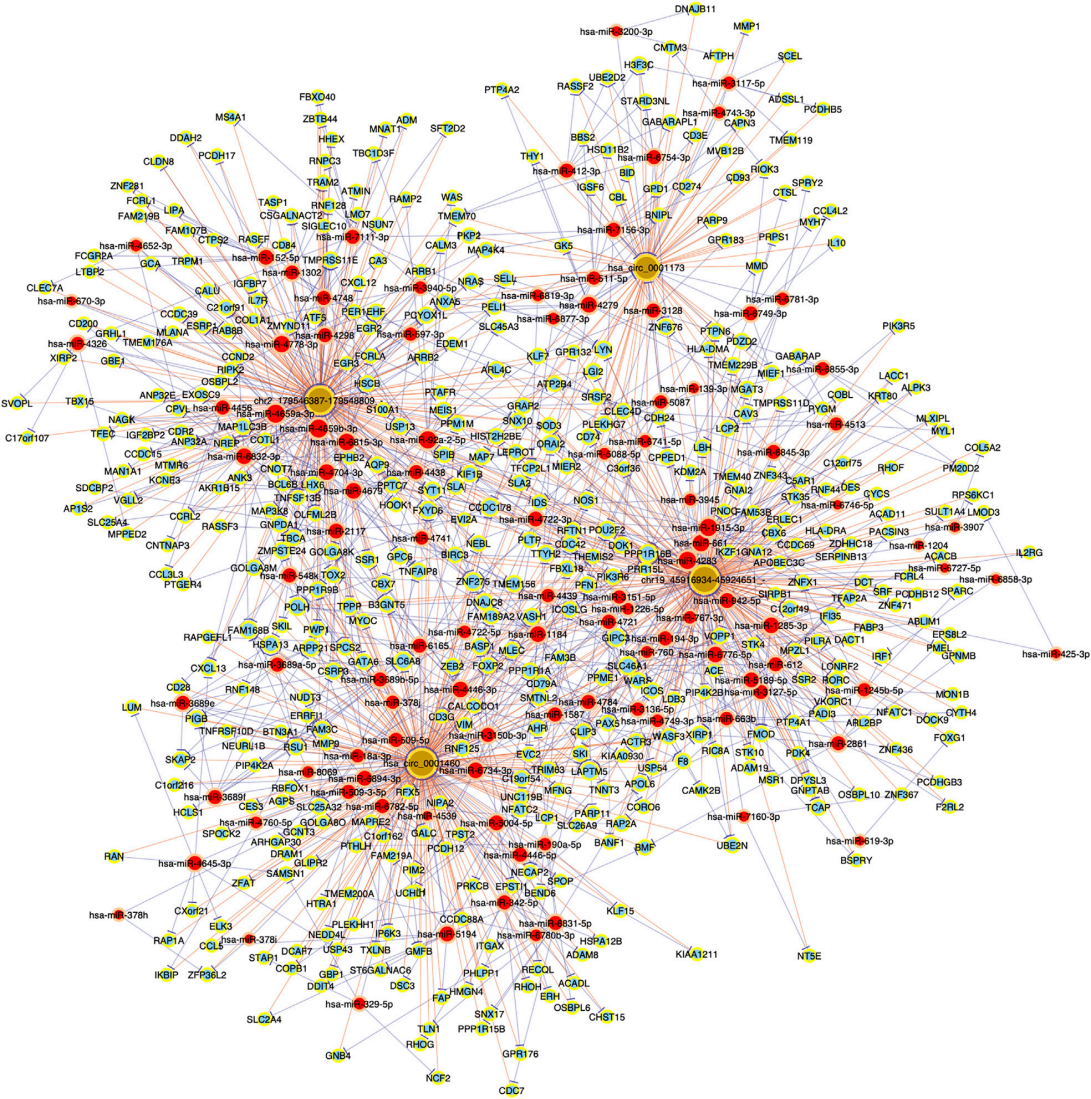
Top 2 up- and top 2 down-regulated circRNAs-associated circRNA-miRNA-mRNA interaction networks in chronic dacryocystitis. Brown round dots represent cirRNAs, red round dots represent miRNAs, light-blue round dots represent mRNAs.

**FIGURE 8:**
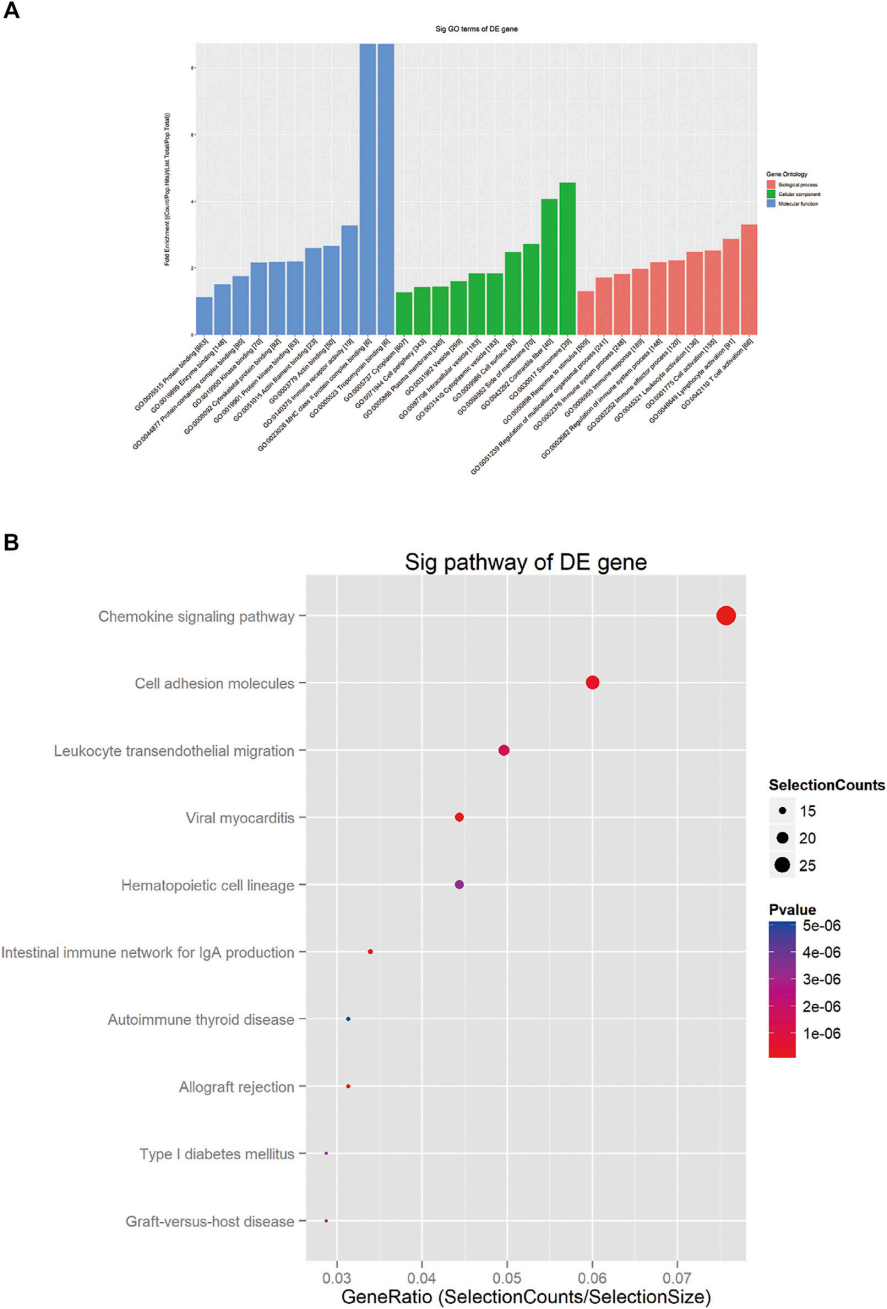
Gene Ontology (GO) and Kyoto Encyclopedia of Genes and Genomes (KEGG) analysis for mRNAs in the circRNA-miRNA-mRNA interaction network. **(A)** The top 30 GO terms of biological process (BP), cellular component (CC) and molecular function (MF) categories for dysregulated mRNAs. **(B)**: The top 10 enriched KEGG pathways for dysregulated mRNAs.

**FIGURE 9:**
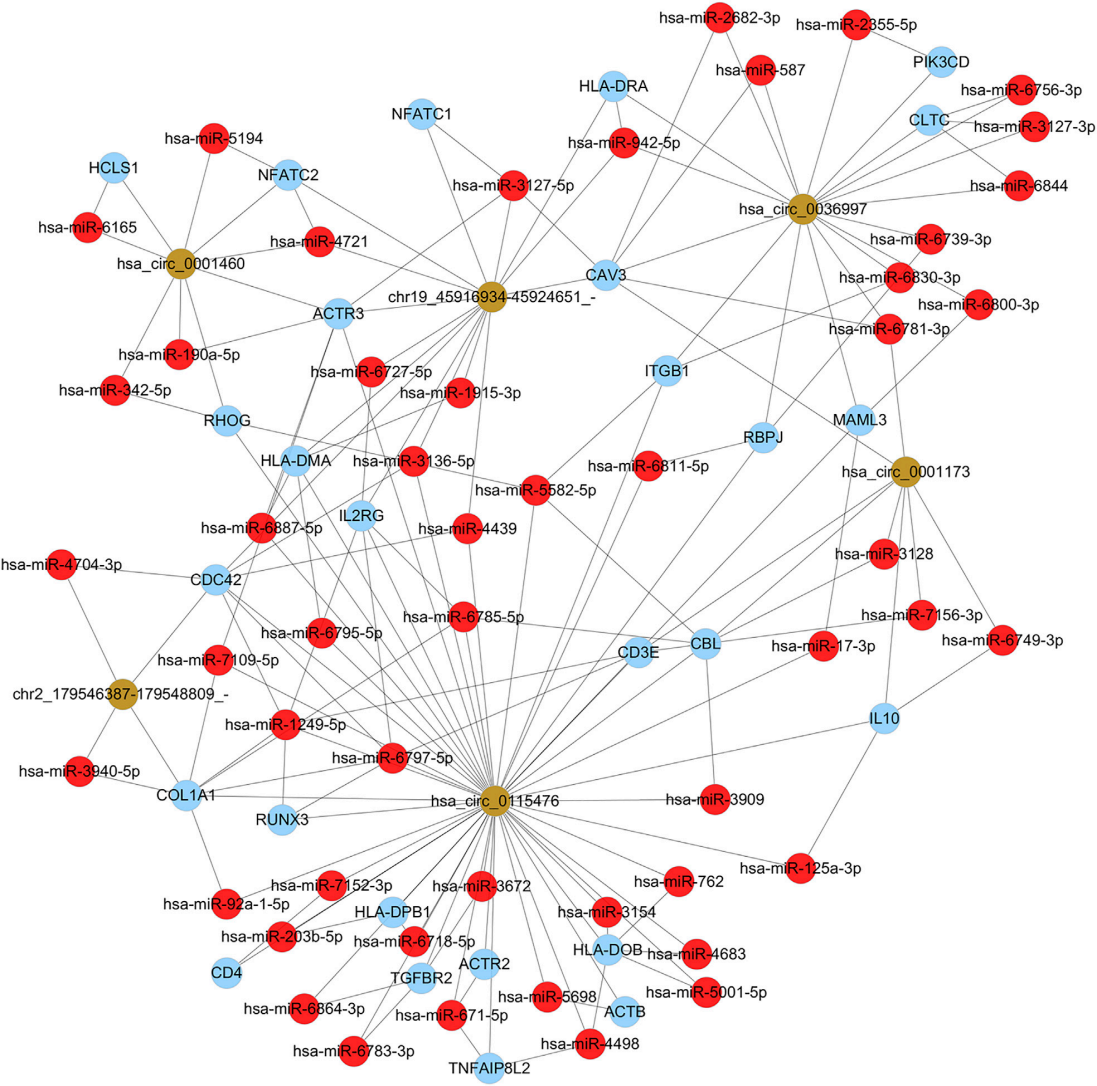
Th1 and Th2 cell differentiation-related circRNA-miRNA-mRNA network. Yellow round dots represent circRNAs, red round dots represent miRNAs, and blue dots represent mRNAs.

To further investigate the role of circRNAs in chronic dacryocystitis, expression correlation between circRNA and mRNA, and ceRNAs (circRNA-miRNA-mRNA) were identified. We first selected the top 3 of up- and 3 of down-regulated circRNAs and investigated miRNAs associated with each circRNA and targeted mRNAs and constructed circRNA-miRNA-mRNA interaction networks. The representative top 2 up-regulated and top 2 down-regulated circRNAs-associated circRNA-miRNA-mRNA interaction networks are shown in Figure 7. Functional analyses were also conducted with differentially expressed mRNAs involved in the networks. As shown in Figure 8A, dysregulated mRNAs were mainly involved in BP of T cell activation, CC of side of membrane and MF of immune receptor activity. According to the results of KEGG, dysregulated mRNAs were associated with autoimmune thyroid disease, chemokine signaling pathway and leucocyte transmembrane migration (Figure 8B). Considering chronic dacryocystitis was closely related to an imbalance of Th1/Th2 status, we then screened out dysregulated mRNAs associated with Th1 and Th2 cell differentiation and constructed Th1 and Th2 cell differentiation-related circRNA-miRNA-mRNA network (Figure 9). There were 28 mRNAs, 74 miRNAs and 6 circRNAs involved in this network. These ceRNA analysis may provide novel underlying mechanism of chronic dacryocystitis.

### Validation for Expression Levels of mRNAs and circRNAs

**FIGURE 10:**
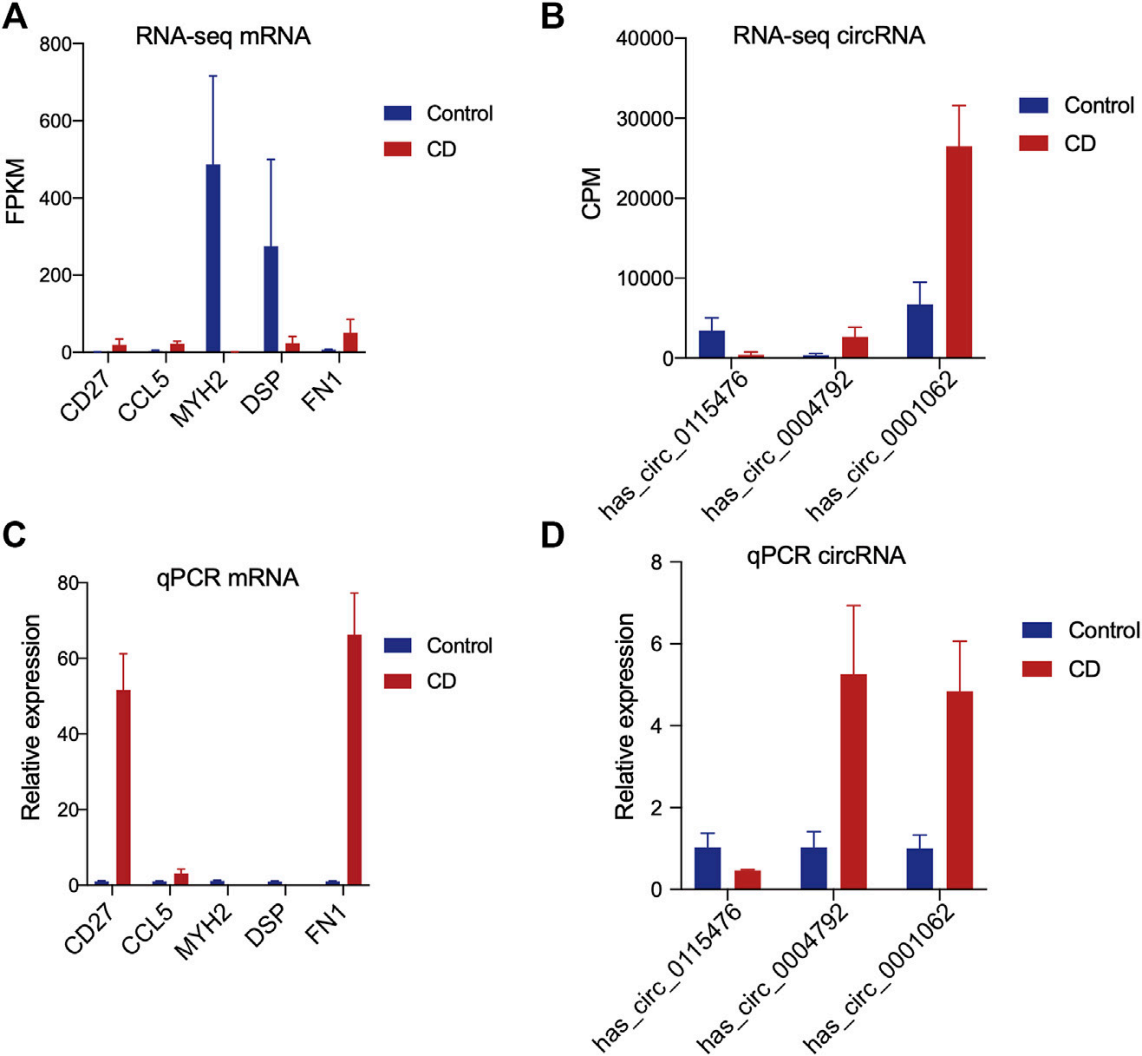
Validation of expression levels of mRNAs and circRNAs in chronic dacryocystitis and control samples. **(A,B)** RNA sequencing analysis of the expression levels of five mRNAs (MYH2, DSP, CD27, CCL5, and FN1) and three circRNAs. **(C,D)** QRT-PCR results of the expression levels of mRNAs and circRNAs detected by in 6 chronic dacryocystitis samples and 6 control samples.

According to the results of RNA sequencing analysis, the MYH2 and DSP were down-regulated, while CD27, CCL5, and FN1 were up-regulated in chronic dacryocystitis samples. The has_circ_0004792 and has_circ_0001062 were up-regulated, whereas has_circ_0115476 was down-regulated in chronic dacryocystitis samples. Moreover, expression levels of these five mRNAs (MYH2, DSP, CD27, CCL5, and FN1), as well as three circRNAs (has_circ_0004792, has_circ_0001062, and has_circ_0115476) in lacrimal sac samples were detected by qRT-PCR. The qRT-PCR results suggested that expression levels of MYH2 and DSP were lower in chronic dacryocystitis samples, and expression levels of CD27, CCL5, and FN1 were significantly higher in chronic dacryocystitis samples. Increased expression of has_circ_0004792 and has_circ_0001062, along with reduced expression of has_circ_0115476 were observed in chronic dacryocystitis samples (Figure 10). Thus, the expression changes of the above 5 mRNAs and 3 circRNAs were validated by qRT-PCR.

## Discussion

Affecting an increasingly growing number of people, chronic dacryocystitis severely disturbs patients’ quality of life. Patients not only suffer from excessive tear production in their daily life but also have increased risks of intraocular infections such as endophthalmitis and corneal ulcer (Teweldemedhin et al., 2017). However, due to the lack of knowledge on its etiology and pathogenesis, there has not been effective etiology therapy for chronic dacryocystitis. In some cases treatment of dacryocystitis could be delayed and orbital cellulitis secondary to dacryocystitis and following abscess formation can even lead to visual compromise (Alsalamah et al., 2018). Thereafter, it’s of great importance to make out the mechanism of the disease and work out more precise etiological therapy. Recently more and more efforts have been made to try to illustrate the process of the disease but studies on specific molecular regulation mechanism of the disease are rare. Recent years, accumulated data have indicated that circRNAs play important roles in a variety of biological process. CircRNAs have multiple molecular mechanisms, such as nuclear transcriptional regulators of host genes, RNA-binding protein sequestering agents or miRNA sponges (Li et al., 2018). Thus dysregulated circRNAs have been reported to be involved in a lot of disease such as osteoarthritis, cancer and many ocular disorders (Zhou et al., 2019; Cui et al., 2020; Zhou et al., 2020; Zou et al., 2020; Zheng et al., 2021). In our study, we performed high-throughput RNA sequencing of the lacrimal sac samples from chronic dacryocystitis patients and control individuals for the first time and constructed circRNA-mRNA and circRNA-miRNA interaction networks trying to throw light on the pathogenesis of chronic dacryocystitis on RNA level.

In this study, we identified 3,909 circRNAs in total, of which 3,199 have been reported in the circBase previously and 710 were novel. Of all these 3,909 circRNAs, 25 circRNAs were significantly differently expressed between chronic dacryocystitis patients and control individuals, including 20 up-regulated and 5 down-regulated. Among the significantly differently expressed circRNAs, hsa_circ_0001173 was the most elevated circRNA in lacrimal sacs of chronic dacryocystitis patient with a fold change of 81.95. Besides, expression levels of hsa_circ_0001460 (fold change = 81.67), hsa_circ_0036997 (fold change = 79.41), hsa_circ_0004087 (fold change = 79.11) were also significantly higher in lacrimal sacs of chronic dacryocystitis patients while expression level of hsa_circ_0115476 (fold change = 0.12) and hsa_circ_0021727 (fold change = 0.04) significantly reduced. The hsa_circ_0001173 has been reported to be significantly upregulated in thymoma and might participate in the abnormal immune regulation of thymus through cell–cell adhesion, MAPK pathway and tumor necrosis factor (TNF) pathway (Wu et al., 2021). The hsa_circ_0004087 has been proved to have the potential of binding Ezrin and activating the AKT pathway and promote colorectal cancer migration (Li et al., 2021). Studies have also shown the dysregulation of hsa_circ_0021727 in peripheral blood mononuclear cells from SLE patients (Luo et al., 2020). Taken together, the dysregulated circRNAs revealed in the lacrimal sacs of the chronic dacryocystitis patients might have potential effect on chronic dacryocystitis. Meanwhile, 1,486 differentially expressed mRNAs including 989 up-regulated and 497 down-regulated were also identified. Three circRNAs (has_circ_0004792, has_circ_0001062, and has_circ_0115476) and five mRNAs (MYH2, DSP, CD27, CCL5, and FN1) were randomly selected, the changes of whose expression levels were validated by qRT-PCR.

To further explore the underlying mechanism of these dysregulated circRNAs, circRNA-mRNA co-expression network and functional analysis for mRNAs in the co-expression network were constructed. The co-expression network was consisted of 5 up-regulated and 3 down-regulated circRNAs. This network indicated hsa_circ_0001173 correlated with up-regulated ITGAM and C5AR1, which according to the GO functional enrichment analysis in our study, associated with the BP of immune system process. In addition, pathway enrichment analysis for up-regulated mRNAs in co-expression network also showed ITGAM and C5AR1 participated in human complement and coagulation cascades. Moreover, pathway enrichment analysis for down-regulated mRNAs revealed CAV3 and CLTCL1 correlated with down-regulated hsa_circ_0115476 took part in bacterial invasion of epithelial cells, which indicated invasion of bacterial and subsequent destruction of lacrimal duct epithelial barrier could be involved in the disease and this was in accordance with previous studies (Eshraghi et al., 2014; Chung et al., 2019). In addition, several muscle fibre-related mRNAs such as TTN, MYL2, and MYOM3 correlated with hsa_circ_0115476 in the circRNA-mRNA co-expression network were downregulated. There are abundant muscle fibers around the lacrimal sac, which participate in tear drainage through the “lacrimal pump” mechanism (Bara et al., 2020). The down-regulation of muscle fibre-related mRNAs might indicate the destruction of the lacrimal pump structure, in which case, tears can’t drain into nose smoothly and this might induce or worsen the tearing and epiphora of chronic dacryocystitis patients. MiRNAs are important post-transcriptional regulators of gene expression which act by direct base pairing to target sites within untranslated regions of mRNAs and miRNA activity has recently been shown to be affected by circRNA acting as miRNA sponges (Li et al., 2020). We then selected the top 3 of up and 3 of down regulated circRNAs and investigated miRNAs associated with each circRNA and targeted mRNA and constructed functional analyses with these mRNAs. The results demonstrated these dysregulated mRNAs were mainly involved in T cell activation and immune system process, which was in accordance with previous reported pathogenesis of chronic dacryocystitis, which suggested imbalance of Th1/Th2 status might play a role in the pathogenesis of chronic dacryocystitis (Yang et al., 2018b). Thus Th1 and Th2 cell differentiation-related circRNA-miRNA-mRNA network was constructed and the results shown 6 circRNAs were involved in this process. The circRNA-miRNA-mRNA interaction network we constructed in this study indicated dysregulated circRNAs could interact with miRNAs to regulate Th1 and Th2 cell differentiation and thus play a role in chronic dacryocystitis. For example, hsa_circ_0115476 could interact with hsa-miR-7152-3p to regulate CD4 expression and this hsa_circ_0115476- hsa-miR-7152-3p- CD4 axis might participate in the CD4^+^ T cell infiltration in chronic dacryocystitis. Our study also has some limitations. Chronic dacryocystitis patients might have a history of applying antibiotic eye drops when first diagnosed with this disease. Although we collected samples only from patients without applying eye drops within 1 month to minimize the influence, their previous use of antibiotic eye drops might have potential effects on the gene expression.

## Conclusion

In conclusion, this study unveils the profiles of mRNAs and circRNAs in the lacrimal sacs from chronic dacryocystitis patients and control individuals for the first time. The results demonstrated that expression levels of circRNAs and mRNAs significantly changed in lacrimal sacs from patients. To further investigate the function of differentially expressed circRNAs, we constructed the circRNA-mRNA co-expression and circRNA-miRNA-mRNA interaction analysis. The results indicated circRNAs might participate in pathogenesis of chronic dacryocystitis through regulating the process of Th1 and Th2 cell differentiation, T cell activation, and bacterial invasion of epithelial cells. The limitation of this study is that most of the results are based solely on bioinformatics models, and other studies will be needed to verify our hypothesis. Taken together, our findings demonstrates that circRNAs are significantly altered in the lacrimal sacs of chronic dacryocystitis patients and provides novel insights for the understanding of the pathogenesis of the disease.

## Data Availability

The datasets presented in this study can be found in online repositories. The names of the repository/repositories and accession number(s) can be found in the article/Supplementary Material.
